# Pleiotropic Anti-Angiogenic and Anti-Oncogenic Activities of the Novel Mithralog Demycarosyl-3D-ß-D-Digitoxosyl-Mithramycin SK (EC-8042)

**DOI:** 10.1371/journal.pone.0140786

**Published:** 2015-11-04

**Authors:** Azahara Fernández-Guizán, Alejandro López-Soto, Andrea Acebes-Huerta, Leticia Huergo-Zapico, Mónica Villa-Álvarez, Luz-Elena Núñez, Francisco Morís, Segundo Gonzalez

**Affiliations:** 1 Department of Functional Biology, IUOPA, Universidad de Oviedo, Oviedo, Spain; 2 Department of Immunology, Hospital Universitario Central de Asturias, Oviedo, Spain; 3 EntreChem S.L., Campus El Cristo, 33006-Oviedo, Spain; Centro Cardiologico Monzino, ITALY

## Abstract

Demycarosyl-3D-ß-D-digitoxosyl-mithramycin SK (DIG-MSK) is a recently isolated analogue of mithramycin A (MTA) that showed differences with MTA in the DNA binding strength and selectivity. These differences correlated with a better therapeutic index and less toxicity in animal studies. Herein, we show that DIG-MSK displays a potent anti-tumor activity against different types of cancer cell lines, ovarian tumor cells being particularly sensitive to this drug. Of relevance, DIG-MSK exerts low toxicity on fibroblasts and peripheral blood mononuclear cells, this toxicity being significantly lower than that of MTA. In correlation with its antitumor activity, DIG-MSK strongly inhibited Sp1-mediated transcription and endogenous *Sp1* mRNA expression, which correlated with the inhibition of the expression of key Sp1-regulated genes involved in tumorigenesis, including *VEGFA*, *BCL2L1* (Bcl-XL), *hTERT*, *BRCA2*, *MYC* and *SRC* in several ovarian cells. Significantly, DIG-MSK was a stronger inhibitor of *VEGFA* expression than MTA. Accordingly, DIG-MSK also exhibited potent anti-angiogenic activity on microvascular endothelial cells. Likewise, it significantly inhibited the gene expression of *VEGFR1*, *VEGFR2*, *FGFR*, *PDGFB* and *PDGFRA* and, additionally, it induced the expression of the anti-angiogenic factors angiostatin and tunstatin. These effects correlated with a pro-apoptotic effect on proliferating microvascular endothelial cells and the inhibition of the formation of endothelial capillary structures. Overall, the pleiotropic activity of DIG-MSK in inhibiting key oncogenic and angiogenic pathways, together with its low toxicity profile, highlight the therapeutic potential of this new drug.

## Introduction

Induction of angiogenesis is a hallmark of the tumorigenic process that plays a key role in tumor progression and metastasis [1]. Cancer cells secrete pro-angiogenic factors that stimulate capillary growth into the tumor bed. Vascular endothelial growth factor (VEGF) is a crucial angiogenic factor that comprises two structurally and functionally related proteins termed VEGFA and VEGFB, being VEGFA the dominant angiogenic factor in most settings [2,3]. VEGF functions as a ligand for two tyrosine kinase receptors: vascular endothelial growth factor receptor 1 (VEGFR1) and 2 (VEGFR2), both expressed on the surface of endothelial cells (ECs). Additionally, a redundant number of angiogenic factors, including angiopoietins, platelet-derived growth factor (PDGF) and fibroblast growth factor (FGF), collaborate with VEGF in the angiogenic process [4].

Anti-angiogenic drugs, mainly targeting VEGF and PDGF, have been successfully introduced in the anti-cancer therapy [5,6]. Bevacizumab, a humanized anti-VEFGA antibody, significantly improved the prognosis of colorectal cancer patients [7]. Multiple inhibitors targeting VEGFR2 and other angiogenic factors, such as sunitinib and sorafenib, displayed promising effects on renal carcinoma patients [5,6]. However, many tumors sooner or later become refractory or resistant to VEGF blockade. This resistance is frequently caused by the activation of alternative angiogenic proteins, such as FGFs, due to preexisting multiple redundant pro-angiogenic molecules and pathways [5,6,8].

**Fig 1:**
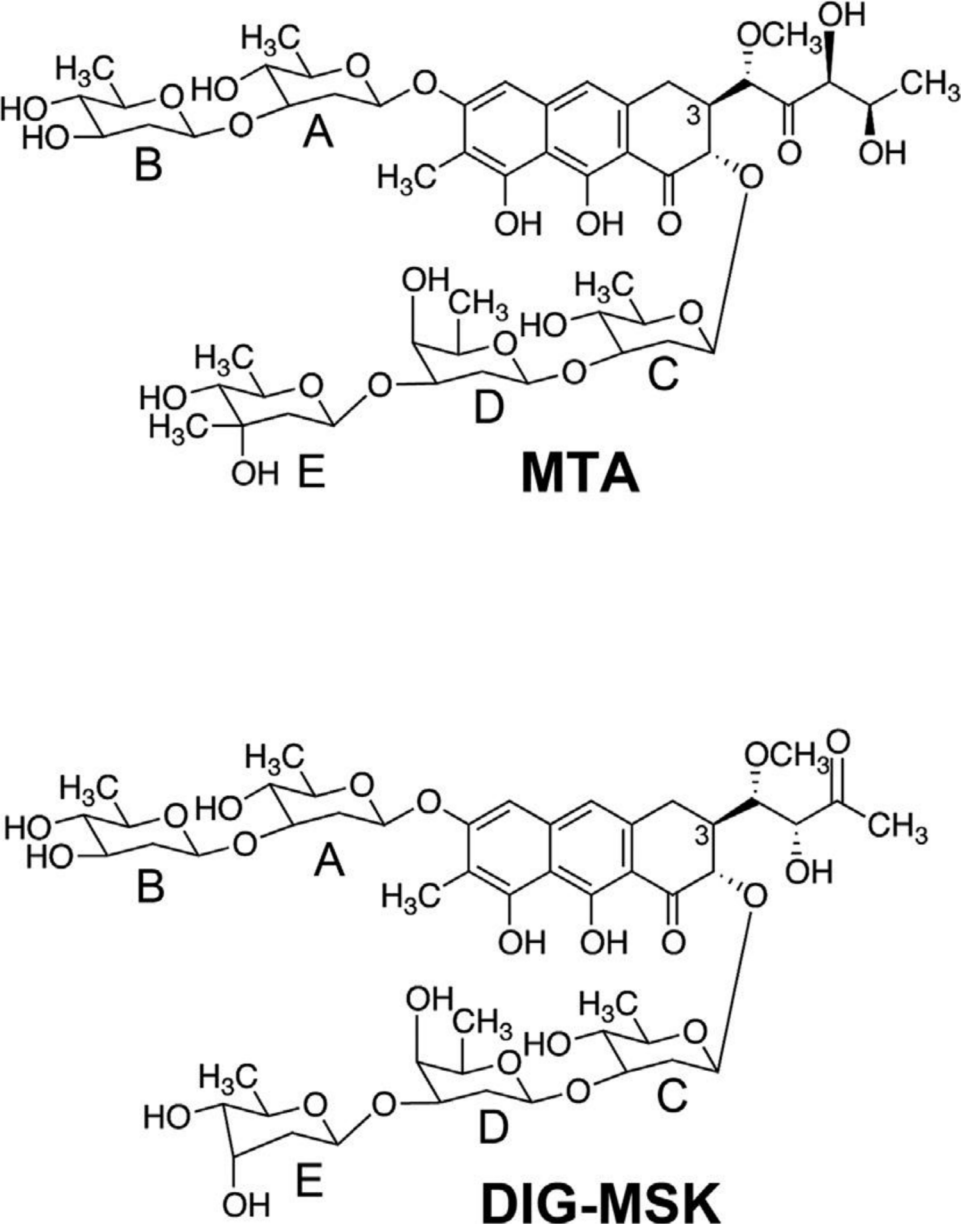
Chemical formulae of mithramycin A (MTA) and demycarosyl-3D-b-D-digitoxosyl-mithramycin SK (DIG-MSK). MTA differs from DIG-MSK in the side chain linked to C-3, and the absence of the methyl group at C-3 on sugar E.

Mithramycin A (MTA) is an aureolic acid-type polyketide antibiotic produced by various species of *Streptomyces* [9]. MTA binds to GC-rich regions in DNA and interferes with the action of several transcription factors; including Sp1. Toxic side effects of MTA have limited its clinical use to the treatment of Paget’s disease and hypercalcemia [10]. Nevertheless, the interest in MTA as an anti-cancer drug has been renewed in 2012, when two clinical trials (NCT01610570 and NCT01624090) were launched at the National Cancer Institute to treat Ewing sarcoma and non-small cell lung cancer. The expansion of mithramycin chemical diversity through combinatorial biosynthesis and biocatalysis has yielded dozens of mithralogs [11]. A promising mithralog with improved therapeutic window, named DIG-MSK (demycarosyl-3D-ß-D-digitoxosyl-mithramycin SK; EC-8042) has been reported (Fig 1). We showed that DIG-MSK is as efficient as MTA in inhibiting Sp1 function, but it differs from MTA in the strength and selectivity of DNA binding [12]. Likewise, DIG-MSK is a stronger inhibitor of endogenous *SP3* gene expression and Sp3-driven transcription than MTA [12]. *In vivo*, the mithralog EC-8042 exerts a potent antitumor activity against ovarian, colon, breast, melanoma, non-small-cell lung and central nervous system cancers, but it is one order of magnitude less toxic than MTA [13].

Sp1 regulates key angiogenic factors, including VEGF, FGF and FGF receptor (FGFR); and the therapeutic inhibition of Sp1 by MTA directly correlated with potent anti-angiogenic effects *in vitro* and *in vivo* [14–18]. Herein, we report that DIG-MSK has a pleiotropic anti-tumor activity, targeting key oncogenic and angiogenic pathways, including those mediated by VEGF, FGF or PDGF. The potent and pleiotropic biological activities exerted by DIG-MSK and its better toxic profile highlight the potential relevance of this agent to treat cancer.

## Materials and Methods

### Mithralogs

MTA and DIG-MSK (EC-8042) were isolated from *Streptomyces argillaceus*, purification was carried out by preparative HPLC and they were identified by HPLC-MS [13]. Their purity was checked by HPLC (~97%). Stock solutions of the different analogues were prepared in DMSO and kept at -20°C until use. This study was approved by the Ethics Committee of the Hospital Universitario Central de Asturias (Spain) and written informed consent was obtained from all donors.

### Cell culture

A2780, OVCAR-3, IGROV-1 SK-OV-3, MCF-7, PANC-1, DLD1, HT-29 and 3T3 cells (all from ATCC) were cultured in DMEM (Lonza). Medium was supplemented with 10% heat-inactivated fetal bovine serum (FBS) (Sigma), 2 mM L-glutamine (Invitrogen), 1 mM pyruvate (Sigma), 100 U/ml penicillin and 100 μg/ml streptomycin (Sigma). HUVEC (ATCC) and HMEC-1 cells (gently provided by Leadartis) were grown in MCDB-131 (Gibco BRL, Rockville, MD) supplemented with 10% heat-inactivated FBS (Invitrogen), 0.01 mg/ml endothelial cell growth supplement (Sigma Aldrich) and 1 ng/ml hydrocortisone. Peripheral blood mononuclear cells (PBMCs) were purified from freshly isolated blood by Ficoll^®^-Paque (Sigma) gradient centrifugation and cultured in RPMI-1640 (Lonza) supplemented with 10% heat-inactivated FBS, 2 mM L-glutamine, 1 mM pyruvate, 100 U/ml penicillin and 100 μg/ml streptomycin.

### SRB assay

The effect of MTA or DIG-MSK on cell viability was determined by using the SRB-method, as described elsewhere [19]. Cell viability was measured after 48 hours of treatment and IC50 values were calculated using the SRB assay carried out in triplicate.

### VEGFR1 and VEGFR2 expression analysis

Cell surface protein expression of VEGFR1 and VEGFR2 was evaluated by flow cytometry by using the following primary antibodies: anti-VEGFR1 (sc-271789, Santa Cruz Biotechnologies) and anti-VEGFR2 (sc-504, Santa Cruz Biotechnologies). After washing, cells were stained with a fluorescein isothiocyanate (FITC)-conjugated secondary antibody (AbD Serotec) and were analyzed by flow cytometry (BD FACScanto, Becton Dickinson).

### Apoptosis and cell cycle analyses

Cells were treated with 200 nM MTA or DIG-MSK and DMSO for 48 hours. Adherent and detached (floating) cell populations were pooled together, and the percentage of cell death by apoptosis and necrosis was determined by flow cytometry (BD FACScanto, Becton Dickinson) after double staining with anti-annexin V antibody (Immunostep) and 7-AminoActinomycin D (7-AAD) (Immunostep). Primary apoptosis was determined as the Annexin-V positive/7-AAD negative cell population. Necrotic cells were characterized as Annexin-V negative/7-AAD positive (primary necrotic cells) and Annexin-V positive/ 7-AAD positive (necrosis arising from apoptotic cells or primary necrosis). Specific cell death was calculated using the following formula: % specific cell death = (% death cell of sample–% death cells of negative control)/(100 - % death cells of negative control) x 100.

The activity of caspases 3 and 9 was measured by using the Caspase 3 Colorimetric Kit (Life Technologies) and the Caspase 9 Fluorimetric Kit (Invitrogen), following the manufacturer’s recommendations.

For cell cycle analysis, cells were treated with MTA (200 nM) or DIG-MSK (200 nM) for various periods of time; cells were harvested, fixed with 70% ethanol, stained with propidium iodide (PI, Sigma) and analyzed by flow cytometry.

### Migration assays

HUVEC and HMEC-1 cells were cultured until convergence and, then, cells were wounded by scratching, rinsed with PBS, and incubated with DMSO, or several doses of MTA or DIG-MSK. Wound closure was monitored over time by using an inverted microscopy (Motic AE2000) and a compatible camera (Motican 1000, Motic). Images were captured at 0, 16, 24, 48 and 72 hours and analyzed with ImageJ software (NCBI). Experiments were made by triplicate and cell migration was expressed as the percentage of distance migrated divided by the length of the initial wound.

### Capillary-like Structure Formation Assay

HMEC-1 cells were incubated in 96 well plates treated with Matrigel, and treated with several doses of MTA or DIG-MSK. After 48 hours, capillary-like structures formation was captured and processed with Angiodraw Software. The angiogenic index (AI) was calculated as the number of branch points in a field. The experiments were made by triplicate and four different fields were counted for each experiment.

### Quantitative Real Time PCR (qRT-PCR)

Total RNA was isolated from cells treated with DMSO, MTA (200 nM), or DIG-MSK (200 nM) for 8 hours using the High Pure RNA Isolating kit (Roche). cDNA was obtained using High Capacity cDNA Reverse Transcription Kit (Invitrogen). Quantitative Real Time PCR (qRT-PCR) was performed in triplicate by using HT7900 RT-PCR and SYBR Green PCR Master Mix (Applied Biosystems). The sequences of the oligos used for real-time PCR are shown in Table 1. The thermal profile was 95°C for 3 minutes, followed by 40 cycles of 95°C for 20 seconds; annealing temperature (specific for each gene, see Table 1) for 30 seconds and 70°C for 30 seconds. Relative expression values of the different genes were calculated from the threshold cycle (Ct) following the 2^−∆∆CT^ method and using *GAPDH* as internal housekeeping control gene for normalization.

**Table 1:** qRT-PCR primers used and thermal profiles.

GENE	FOWARD PRIMER	REVERSE PRIMER	T°
***C-MYC***	5´-GGTCTCCCCTACCCTCTCAACGA-3´	5´-GGCAGCAGGATAGTCCTTCCGAGT-3´	55°C
***C-SRC***	5´-GTCTGACTTCGACAACGCCAAG-3´	5´-CCCCTTGAGAAAGTCCAGCAA-3´	55°C
***TERT***	5´-TGAACTTGCGGAAGACAGTG-3´	5´-GGGTTCTTCCAAACTTGCTG-3´	60°C
***BCL-XL***	5´-GAAGGGACTGAATCGGAGATGGAGAC-3´	5´-AGAGTGGATGGTCAGTGTCTGGTCAT-3´	55°C
***BRCA2***	5´-CGTACACTGCTCAAATCATTC-3´	5´-GACTAACAGGTGGAGGTAAAG-3´	60°C
***MICA***	5´-CACCTGCTACATGGAACACAGC-3´	5´-TATGGAAAGTCTGTCCGTTGACTCT-3´	60°C
***P21***	5´-GGAAGACCATGTGGACCTGT-3´	5´-AAGATGTAGAGCGGGCCTTT-3´	60°C
***FGF***	5´-CAGCTGTACAAGAACAGAGGCTTTC-3´	5´-AAATGGGTCCATGCTGTCGGTCTCC-3´	60°C
***FGFR***	5´-CATCCGCTGGCTTAAGGATGGAC-3´	5´-ATCACGAGACTCCAGTGCTGATG-3´	60°C
***PDGFA***	5´- CCCCTGCCCATTCGGAGGAAGAG-3´	5´-TTGGCCACCTTGACGCTGCGGTG-3´	62°C
***PDGFB***	5´-GATCCGCTCCTTTGATGATC-3´	5´-GTCTCACACTTGCATGCCAG-3´	60°C
***PDGFRA***	5´-ATCAATCAGCCCAGATGGAC-3´	5´-TTCACGGGCAGAAAGGTACT-3´	60°C
***PDGFRB***	5´-AATGTCTCCAGCACCTTCGT-3´	5´-AGCGGATGTGGTAAGGCATA-3´	58°C
***EGF***	5´-TGCCAACTGGGGGTGCACAG-3´	5´-CTGCCCGTGGCCAGCGTGGC-3´	60°C
***VEGFA***	5´-AGGAGGAGGGCAGAATCATCA-3´	5´-CAGGGATTTTCTTGTCTTG-3´	62°C
***VEGFB***	5´-AGCCAGTGTGAATGCAGA-3´	5´-ATAGCCTCTGAGGCAAGT-3´	62°C
***VEGFC***	5´-AGGCCACGGCTTATGCAA-3´	5´-TAGACATGCATCGGCAGGAA-3´	62°C
***VEGFD***	5´-CATCTCAGTCCACAT TGG-3´	5´-GGCAAGCACTTACAACCT-3´	62°C
***VEGFR1***	5´-AAAAACAACCACAAAATACAACAA-3´	5´-TCTTAATGCCAAATGCTGATGCTT-3´	50°C
***VEGFR2***	5´-ACGTCTGGTCTTTTGGTGTTTTGC-3´	5´-ATACTGACTGATTCCTGCTGTGTT-3´	50°C
***VEGFR3***	5´-CCTGAAGAAGATCGCTGTTC-3´	5´-GAGAGCTGGTTCCTGGAGAT-3´	65°C
***HIF1A***	5´-TGCTTGGTGCTGATTTGTGA-3´	5´-GGTCAGATGATCAGAGTCCA-3´	68°C
***ENDOSTATIN***	5´-ATGCTGACATTCACCTGCC-3´	5´-ATGAAGTCAGCACCTGCTGG-3´	60°C
***ANGIOSTATIN***	5´-AATTCCATGTGCAAGACTGGGAATGGAA-3´	5´-TTGAATTCTTAACAGGACGGTATCTTACA-3´	60°C
***TSP1***	5´-CCTCAGGAACAAAGGCTGCTC-3´	5´-GCCAATGTAGTTAGTGCGGATG-3´	60°C
***TUMSTATIN***	5´-TTAAAGGGAAATCCTGGTGAC-3´	5´-GTTCTGGTTTCTTTGATTTCG-3´	60°C
***GAPDH***	5´-CGGAGTCAACGGATTTGGTC-3´	5´-ATCATATTGGAACATGTAAACCATGTAG-3´	50–62°C

### ELISA measurements of secreted VEGF in culture supernatants

A2780, OVCAR-3 and IGROV-1 cells were cultured in 6 well plates (1x10^6^ cells/well) and treated with MTA (200 nM), DIG-MSK (200 nM) or DMSO for 72 hours. Supernatants were collected and VEGF level was measured by quantitative VEGF enzyme-linked immunosorbent assay (ELISA) (Sigma Aldrich), according to the manufacturer's instructions.

### Statistical analysis

Continuous variables were compared with Mann-Whitney U test. The p values <0.05 were considered statistically significant.

## Results

### Effect of DIG-MSK on the toxicity and the cell cycle of cancer cell lines and mononuclear blood cells

The effect of MTA and DIG-MSK on the viability of a panel of cell lines was analyzed by determining the drug concentrations required for inhibiting cell viability by 50% (IC50) [19]. MTA and DIG-MSK strongly reduced the viability of most of the cell lines analyzed at low nanomolar doses (Table 2). Lower concentrations of DIG-MSK than MTA were needed to obtain equitoxic effects on A2780 cells (2.84 nM vs. 13.75 nM; p<0.01) IGROV-1 cells (7.5 nM vs. 136 nM; p<0.001) and MCF-7 cells (17.26 nM vs. 99.36 nM; p<0.001). Nevertheless, MTA was more toxic than DIG-MSK on PANC-1 cells (Table 2). Of note, both drugs were particularly efficient on ovarian cells, in which low nanomolar doses successfully inhibited cell viability. Conversely, the toxicity of these analogues in embryo fibroblasts 3T3 was significantly lower than in cancer cell lines.

**Table 2:** Cytotoxic effect (IC_50_) of MTA and DIG-MSK against cancer cell lines and endothelial cell lines.

CELL LINE	Cellular type	MTA	DIG-MSK	P
**A2780**	Ovarian cancer	13.75 nM	2.84 nM	<0.01
**OVCAR-3**	Ovarian cancer	3 nM	6.75 nM	Ns
**IGROV-1**	Ovarian cancer	136 nM	7.5 nM	<0.001
**SK-OV-3**	Ovarian cancer	10.50 nM	16.23 nM	Ns
**MCF-7**	Brest cancer	99.36 nM	17.26 nM	<0.001
**PANC-1**	Pancreatic cancer	33.83 nM	434.7 nM	<0.001
**DLD1**	Colorectal cancer	209.9 nM	180 nM	Ns
**HT-29**	Colorectal cancer	1.2 uM	2.56 nM	Ns
**HMEC-1**	Umbilical vein endothelial cell	7 nM	8 nM	Ns
**HUVEC**	Umbilical vein endothelial cell	39.87 nM	47.15 nM	Ns
**3T3**	Mouse embryo Fibroblast	2.56 μM	2.19 μM	Ns

Ns: Non significant

**Fig 2:**
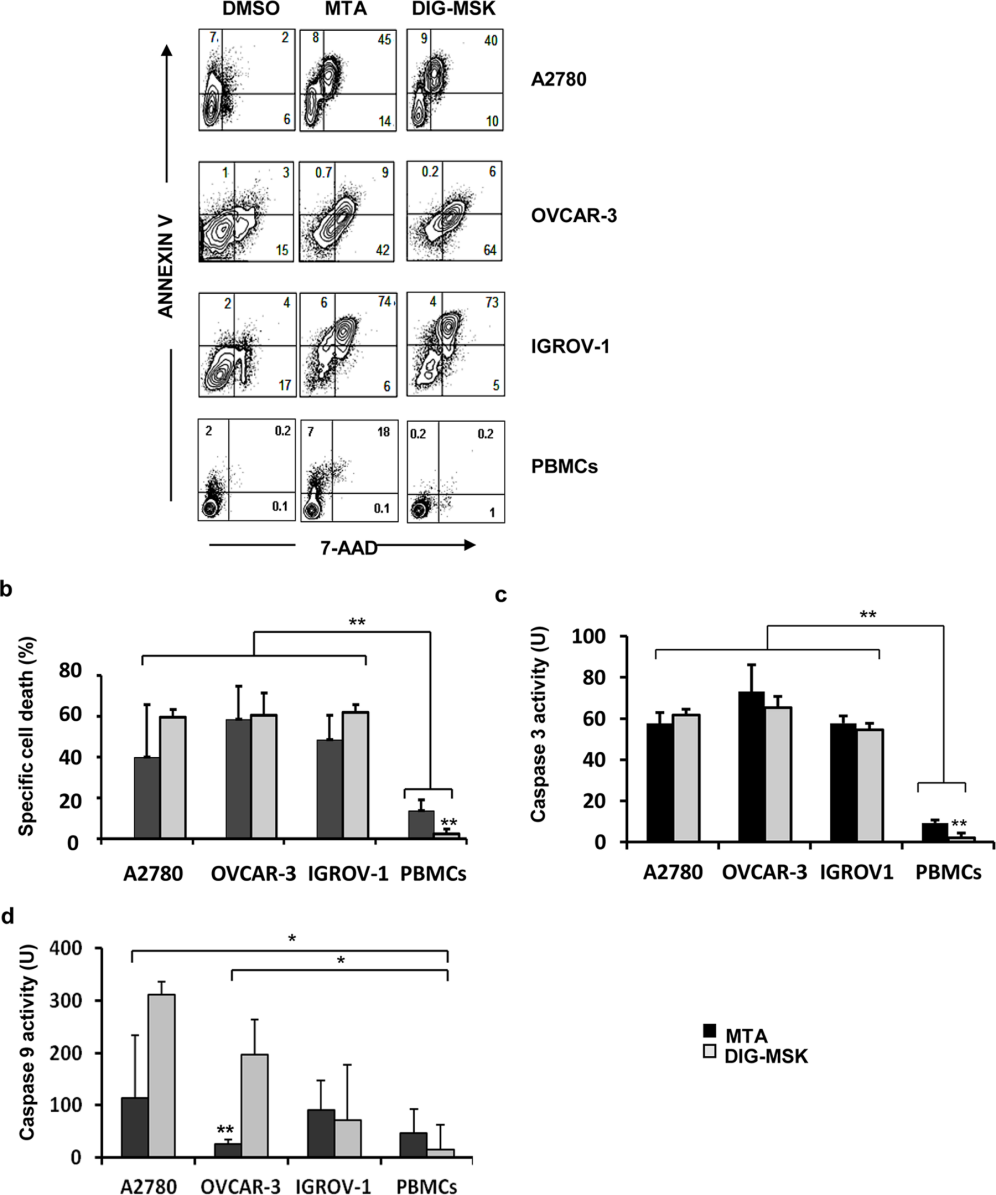
Pro-apoptotic activity of MTA and DIG-MSK in ovarian and mononuclear blood cells. a) Ovarian cells and PBMCs were treated with MTA (200 nM), DIG-MSK (200 nM) or DMSO and cell death was analyzed by flow cytometry by staining the treated cells with Annexin-V-FITC and 7-AAD. b) The bars represent the mean ± SEM of the specific cell death of at least three independent experiments. PBMCs were obtained from six unrelated donors. c and d) Analysis of caspase-3 and -9 activities in ovarian cells and PBMCs treated with 200 nM MTA or DIG-MSK compared to vehicle (DMSO) treated cells. The bars represent the mean ± SEM of the units (U) of caspase activity obtained from at least three independent experiments (*p<0.05; **p<0.01, Mann-Whitney U test).

The toxic effect of these drugs on the ovarian cells was further analyzed by measuring apoptosis (Fig 2A and 2B). Ovarian cells were treated with 200 nM MTA or DIG-MSK and cell death was analyzed by annexin V staining. A strong cytotoxic effect was observed with both analogues in the three cell lines analyzed, causing cell death by both apoptosis and necrosis (ranging from 40% to 60%). Of note, the induction of apoptosis in PBMCs obtained from 6 healthy donors was very low (Fig 2B), causing DIG-MSK significantly lower toxicity than MTA (2% vs. 12%; p<0.001). To extend these observations, caspase 3 and caspase 9 activities were analyzed. Both drugs induced a significant increase in caspase 3 and 9 activities in ovarian cells (Fig 2C and 2D). In agreement with the cell death, both MTA and DIG-MSK had a significantly lower cytotoxic effect on healthy PBMCs than in ovarian cells, although the caspase 3 activity induced by DIG-MSK was significantly lower than MTA (p<0.01).

**Fig 3:**
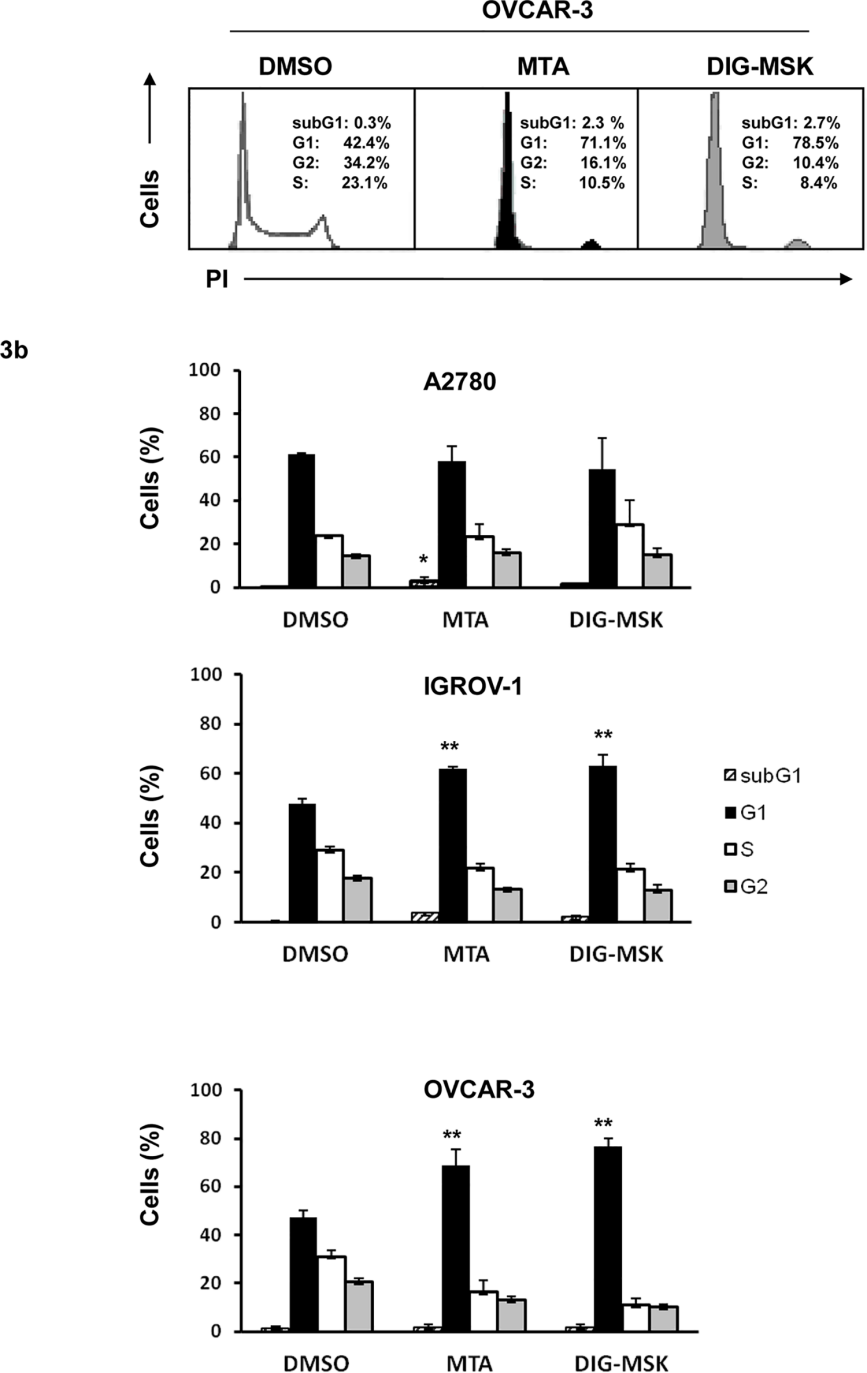
Cell cycle distribution in ovarian cancer cells treated with MTA and DIG-MSK. a) Ovarian tumor cells were treated with MTA (200 nM), DIG-MSK (200 nM) or DMSO, and the cell cycle distribution was analyzed by propidium iodide (PI) staining and flow cytometry analysis. Representative histograms of OVCAR-3 cells are shown. b) The bars represent the mean ± SEM of the percentage of cells in the different phases of the cell cycle in IGROV-1, A2780 and OVCAR-3 ovarian carcinoma cells. The results are obtained from at least three independent experiments (drug vs. DMSO; *p<0.05; **p<0.01, Mann-Whitney U test).

Next, we analyzed the effect of MTA and DIG-MSK on the proliferation of ovarian tumor cells by analyzing the cell cycle distribution. As shown in Fig 3, both analogues halted the cell cycle and induced a G1 arrest in IGROV-1 and OVCAR-3 cells, whereas no such effect was observed in A2780 cells.

### DIG-MSK inhibits the expression of Sp1-target genes in ovarian cancer cells

**Fig 4:**
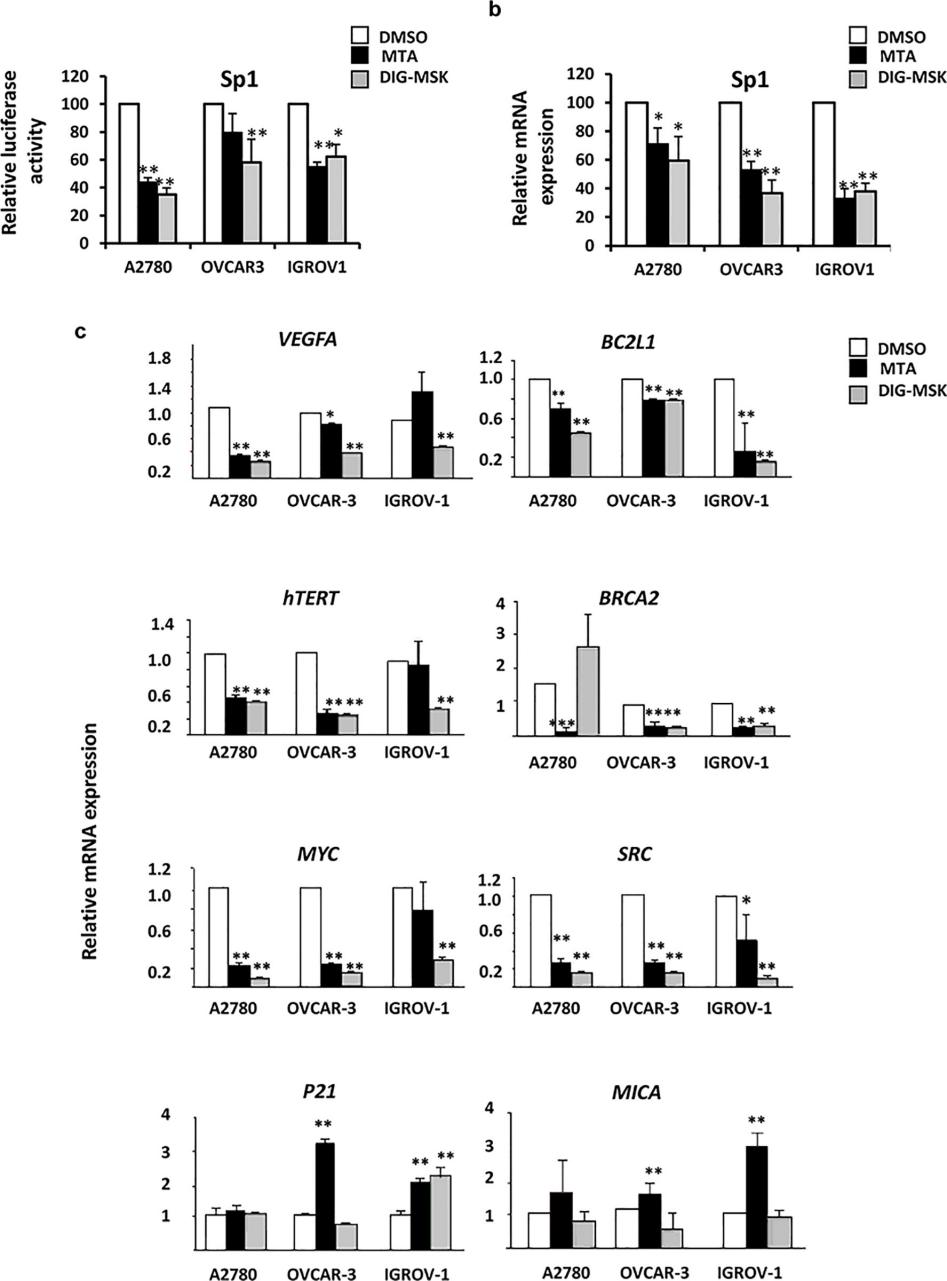
DIG-MSK regulates the expression of *SP1* and key oncogenic genes in ovarian tumor cells. (a) Relative changes in luciferase activity of a transfected Sp1-reported vector in the presence of 200 nM MTA or DIG-MSK compared to untreated ovarian cancer cells. qRT-PCR analysis of the expression of *SP1* gene and several key Sp1-regulated oncogenic genes in ovarian cancer cells treated or untreated with 200 nM MTA or DIG-MSK. Data represent the mean ± SEM of Ct values obtained from at least three independent experiments made by duplicate. The relative mRNA expression was obtained by comparison of the expression profiles of untreated cells (DMSO) versus treated ones (*p<0.05; **p<0.01, Mann-Whitney U test).

To get further insight into the signaling pathways underlying the activity of MTA and DIG-MSK, the effect of these compounds on the expression of a transfected luciferase reporter vector containing several Sp1-response elements was determined. Both drugs were potent inhibitors of Sp1-mediated transcriptional activity in ovarian cells (Fig 4A). In agreement, they were also potent inhibitors of endogenous *Sp1* expression and the transcription of eight Sp1-target genes involved in key aspects of the ovarian tumorigenesis as determined by qRT-PCR (Fig 4B and 4C). MTA and DIG-MSK significantly down-regulated the transcript levels of *VEGFA*, *BCL2L1* (Bcl-XL), *hTERT*, *BRCA2*, *MYC* and *SRC* genes. DIG-MSK showed, in general, a similar or stronger inhibitory effect than MTA on the transcription of such Sp1-regulated genes. Particularly, DIG-MSK was more efficient than MTA in inhibiting *VEGFA* gene expression in A2780 cells (1.3-fold), OVCAR-3 cells (2.8-fold, p<0.05) and IGROV-1 (3.2-fold, p<0.001) cells (Fig 4C). However, a stronger induction of *P21* and *MICA* expression was observed upon treatment with MTA, the latter being a gene involved in the activation of the anti-tumor immune response [20].

### DIG-MSK exerts a pro-apoptotic effect and suppresses tube formation by microvascular endothelial cells

**Fig 5:**
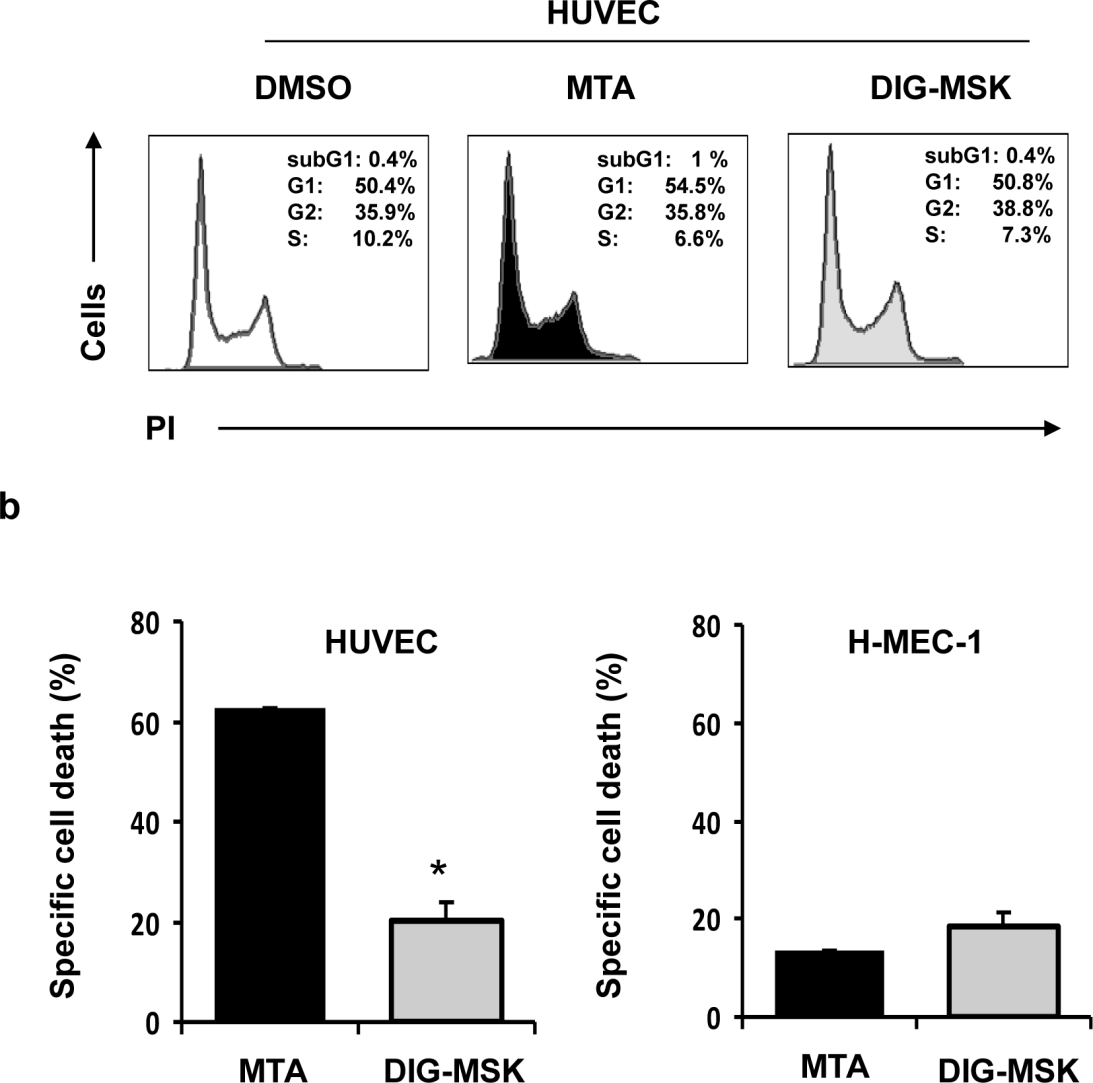
Cell cycle distribution and pro-apoptotic effect on microvascular endothelial cells upon exposure to MTA and DIG-MSK. a) HUVEC and HMEC-1 cells treated with MTA (200 nM) or DIG-MSK (200 nM) or DMSO were stained with propidium iodide (PI) and the cell cycle distribution was analyzed by flow cytometry. A representative cytometric profile of HUVEC cells is shown. b) Cell death was analyzed by flow cytometry in ECs (HUVEC and HMEC-1 cells) treated with 200 nM MTA, DIG-MSK or in untreated cells after staining them with Annexin-V and 7-AAD. The bars represent the mean ± SEM of the specific cell death. At least three independent experiments were analyzed (*p<0.05; Mann-Whitney U test).

To further analyze the anti-angiogenic properties of DIG-MSK, its effect on microvascular endothelial cells (ECs) was analyzed. No significant effect was observed on cell cycle progression in HUVEC or HMEC-1 cells (Fig 5A). Likewise, no EC migration was not significantly affected by DIG-MSK, as determined by the wound/scratch-healing assay (not shown). However, DIG-MSK treatment induced significantly lower levels of cell death in HUVEC cells than MTA, with no marked differences observed in HMEC-1 cells (Fig 5B).

**Fig 6:**
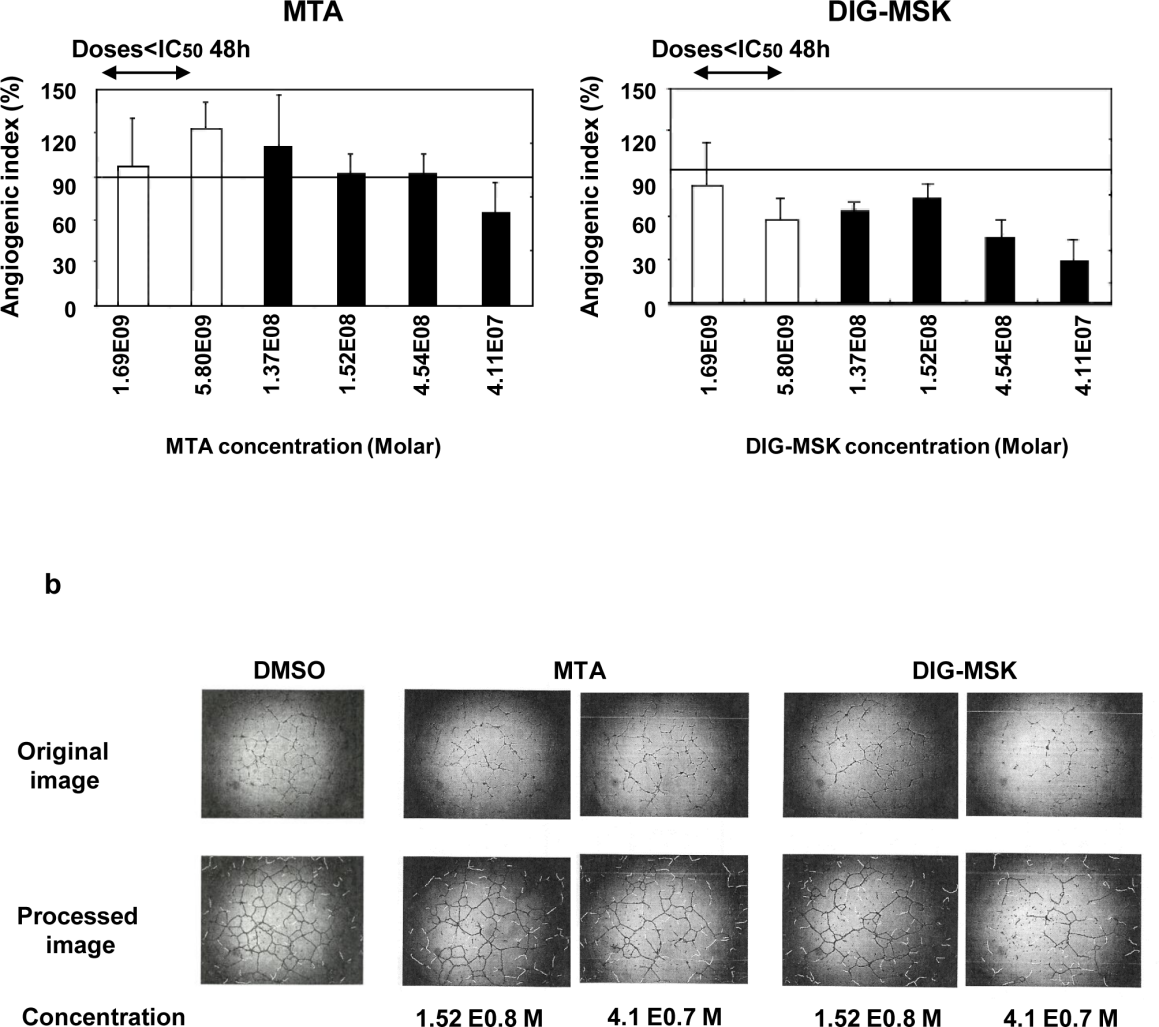
Effect of MTA and DIG-MSK on tube formation by human microvascular endothelial cells. a) HMEC-1 cells were seeded onto Matrigel-coated wells and incubated with DMSO or various concentrations of MTA and DIG-MSK for 48 hours. Capillary-like structures formation was captured and processed with Angiodraw Software. Angiogenic index was calculated as the number of branch points in a field. The bars represent the mean ± SEM of the angiogenic index of four independent experiments (*p<0.05; Mann-Whitney U test). b) Representative appearance of HMEC-1 tube formation. Untreated control cells and cells treated with MTA and DIG-MSK are presented.

Next, the formation of new blood vessel was analyzed by conducting the endothelial tube formation assay in which Matrigel was used as a substrate. The integrity of the capillary-like structure formed by HMEC-1 cells was significantly inhibited by DIG-MSK in a dose-dependent manner, and this effect was observed at non-cytotoxic doses (Fig 6 and S1 Fig). However, MTA was only able to inhibit capillary-like formation at high doses, which was associated with a high toxicity in HMEC-1 cells.

### MTA and DIG-MSK modulate the expression of angiogenic genes in human microvascular endothelial cells

**Fig 7:**
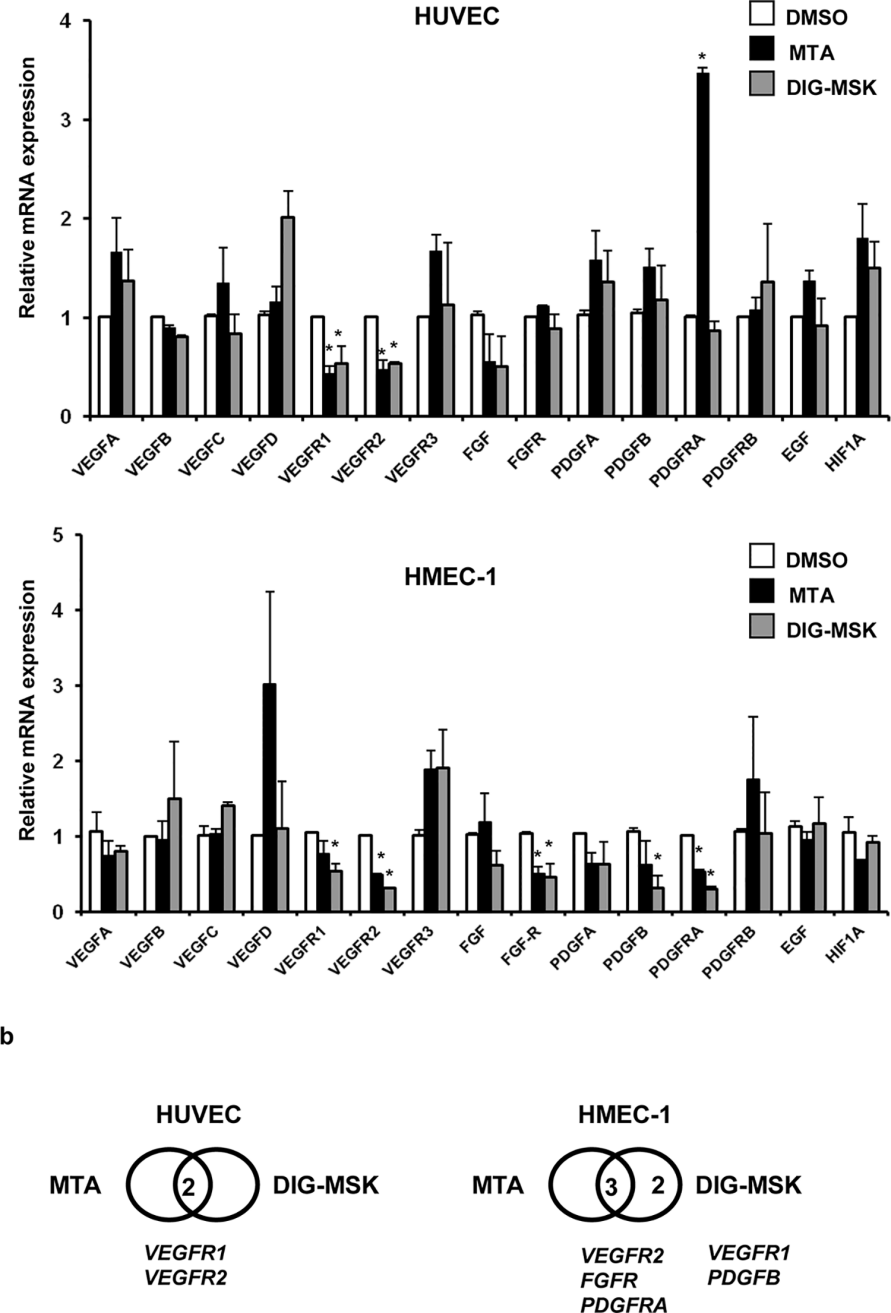
MSK and DIG-MSK modulate the expression of key angiogenic genes in ovarian carcinoma cells. a) qRT-PCR analysis of the expression of several key angiogenic genes in the presence of 200 nM MTA or DIG-MSK compared with untreated ECs. Data represent the mean ± SEM of Ct values obtained from at least three qRT-PCR independent experiments made by duplicate. The relative mRNA expression was obtained by comparison of the expression profiles of untreated cells (DMSO) versus treated ones (*p<0.05; **p<0.01; Mann-Whitney U test). b) Venn diagrams representing genes down-regulated by treatment with MSK or DIG-MSK (p<0.05). Numbers inside the intersections correspond to genes repressed by both treatments.

**Fig 8:**
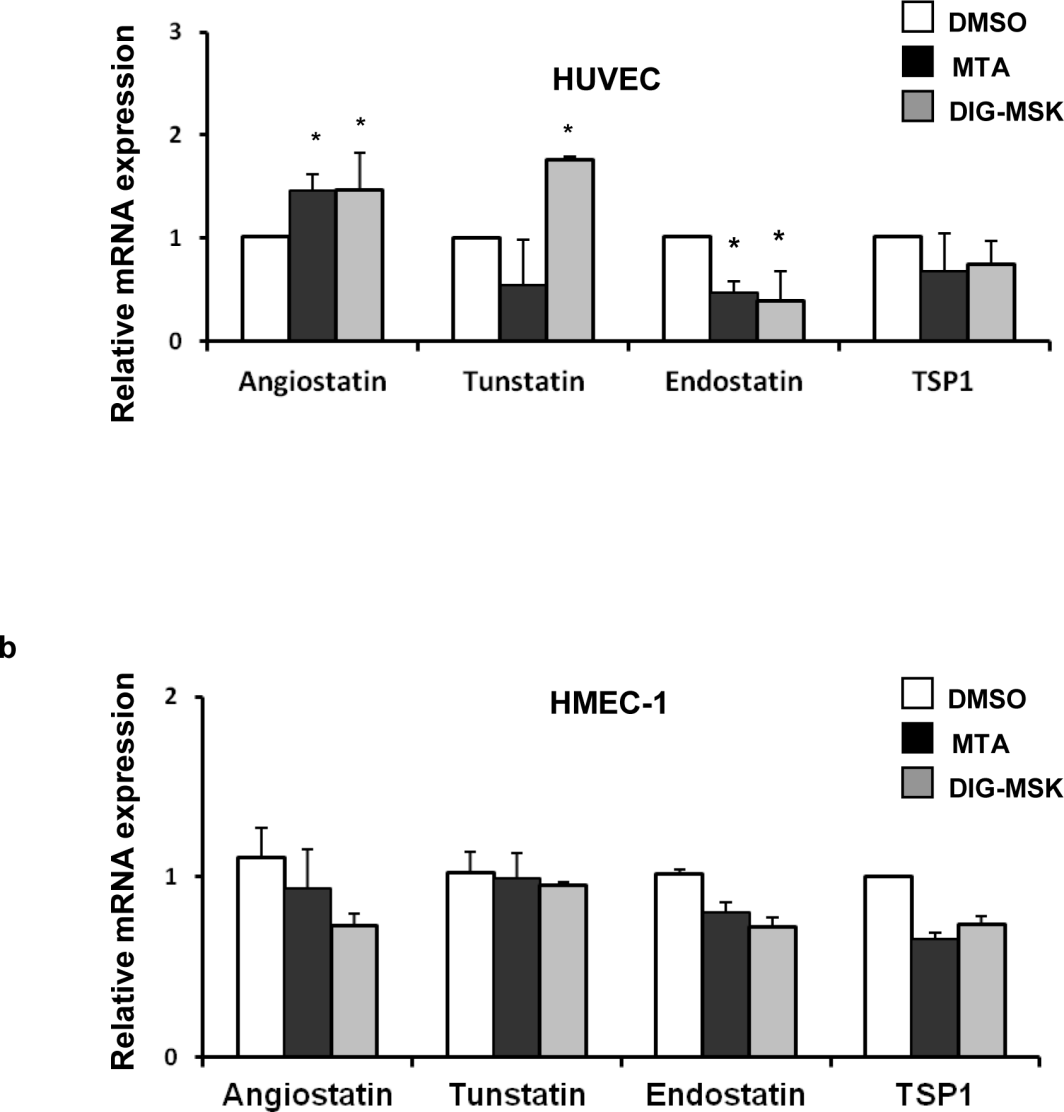
Analysis of the expression of key anti-angiogenic genes in ECs treated with MTA or DIG-MSK. qRT-PCR analysis of the expression of several key anti-angiogenic genes in the presence of 200 nM MTA or DIG-MSK compared to untreated ECs. Data represent the mean ± SEM of Ct values obtained from at least three qRT-PCR independent experiments made by duplicate in HUVEC (a) or HMEC-1 (b) endothelial cells. The relative mRNA expression was obtained by comparison of the expression profiles of untreated cells (DMSO) versus treated ones (*p<0.05; Mann-Whitney U test).

To gain further insight into the mechanism underpinning the suppression of capillary formation by DIG-MSK, the impact of this drug on the transcription of key angiogenic genes (*VEGFA*, *VEGFB*, *VEGFC*, *VEGFD*, *VEGFR1*, *VEGFR2*, *VEGFR3*, *FGF*, *FGFR*, *PDGFA*, *PDGFB*, *PDGFRA*, *PDGFRB*, *EGF and HIF1A*) in ECs was analyzed by qRT-PCR. As shown in Fig 7A, a significant reduction in *VEGFR1* and *VEGFR2* gene expression in HUVEC cells (ranging from 40 to 50% of inhibition). A down-regulation of *VEGFR2*, *FGFR* and *PDGFRA* gene expression was observed with both analogues in HMEC-1 cells. DIG-MSK also inhibited *VEGFR1* and *PDGFB* expression in these cells (Fig 7A and 7B). Contrarily, MTA significantly up-regulated the angiogenic factor *PDGFA* in HUVEC cells. Similarly, their effect on the transcript levels of key anti-angiogenic genes encoding *angiostatin*, *tunstatin*, *endostatin* and *thrombospondin-1* was analyzed (Fig 8). *Angiostatin* gene expression was up-regulated by both analogues and *tunstatin* was induced by DIG-MSK in HUVEC cells. Conversely, a reduction of *endostatin* expression was observed in HUVEC cells treated with both drugs.

### Effect of MTA and DIG-MSK on the secretion of VEGF and the surface expression of VEGFR1 and VEGFR2

**Fig 9:**
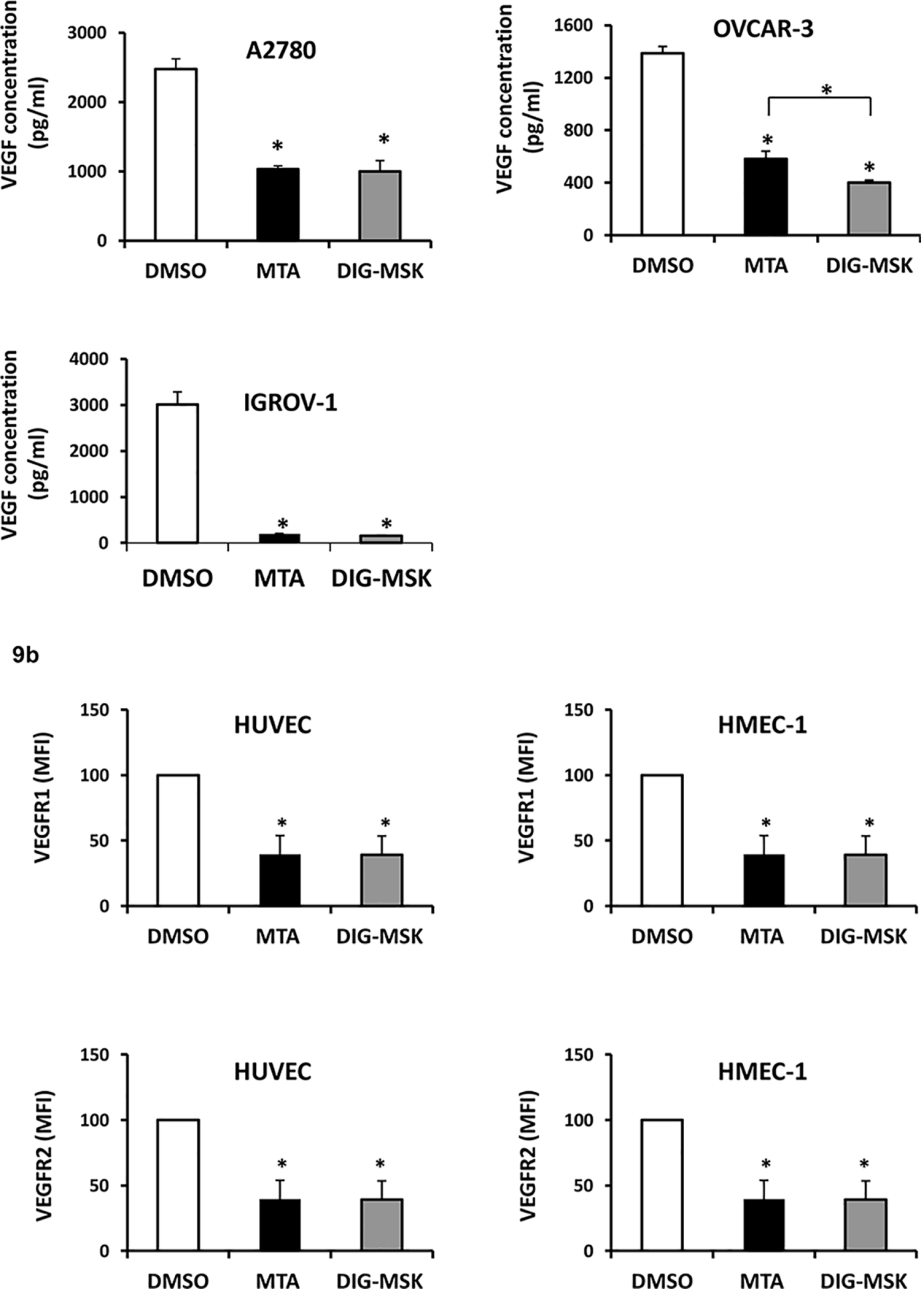
Effect of MTA and DIG-MSK on the secretion of VEGF and the surface expression of VEGFR1 and VEGFR2. a) Soluble VEGF levels were measured by ELISA in supernatants of ovarian carcinoma cells (A2780, OVCAR-3 and IGROV-1) treated with 200 nM MTA or DIG-MSK compared with untreated cells. b) The surface expression of VEGFR1 and VEGFR2 was analyzed by flow cytometry in ECs treated with 200 nM MTA or DIG-MSK relative to DMSO treated cells. Data represent the mean ± SEM of levels obtained from at least three independent experiments. (*p<0.05; Mann-Whitney U test).

Given the strong inhibition of *VEGF* gene expression in ovarian cancer cells, and the reduction of the mRNA expression of VEGF receptors observed in ECs upon exposure to MTA and DIG-MSK, we next studied the modulation of these key regulators of the angiogenic process at protein level under these conditions. ELISA analyses demonstrated that both drugs strongly inhibited the secretion of VEGFA in ovarian tumor cells (A2780 cells; MTA: 2.39-fold, DIG-MSK: 2.55-fold, p<0.05; OVCAR-3 cells; MTA: 2.40-fold, DIG-MSK: 3.44-fold, p<0.05) (Fig 9A). Further, both drugs completely inhibited the production of VEGFA in IGROV-1 cells (15.4-fold MTA, 19.63-fold DIG-MSK, p<0.05). Of note, in OVCAR-3 cells, DIG-MSK was significantly more efficient than MTA in inhibiting the secretion of VEGFA (1.45-fold, p<0.05). Likewise, flow cytometry analyses also showed that surface expression of VEGFR1 and VEGFR2 was significantly down-regulated by MTA and DIG-MSK in HUVEC and HMEC-1 cells (p<0.01) (Fig 9B).

Overall, our results show that DIG-MSK has a pleiotropic anti-angiogenic effect by up-regulating the expression of key angiogenic genes as well as repressing the transcription of key anti-angiogenic genes, indicating that this new drug may be useful for anti-cancer therapies that target angiogenic pathways.

## Discussion

MTA is a DNA-binding drug that shows preference for G/C-rich DNA sequences, and, therefore, a significant part of the anti-tumor activity of MTA is thought to be mediated by the blockade of the binding of transcription factors such as Sp1 [21]. Sp1 expression and activity are up-regulated in many cancers, correlating with advanced stage and poor prognosis [22–26]. Accordingly, Sp1 inhibition may decrease tumor formation, growth and metastasis [14,27,28], representing an attractive target, despite being considered undruggable by conventional drug discovery approaches [29].

DIG-MSK is a novel analogue of MTA that shares the general features of other mithralogs, such as the preference for binding to G/C-rich DNA sequences and inhibition of Sp1-driven transcription [21]. Nevertheless, DIG-MSK has significant differences in the strength and specificity of DNA binding [12]. Thus, DIG-MSK is a stronger inhibitor of the expression of Sp3 and Sp3-driven transcription than MTA. In spite of the fact that Sp1 and Sp3 recognize the same DNA element with similar affinity, these two transcription factors possess different functions, and the relative level of both proteins differentially regulates the expression of several genes [22,30, 31,32]. Overall, these differences between MTA and DIG-MSK point that different therapeutic activity and toxicity between both analogues may exist. In fact, initial studies reported that DIG-MSK has an improved therapeutic index compared to the parental MTA, since it is one order of magnitude less toxic than MTA *in vivo* [13]. In agreement, in our study we report that DIG-MSK displays low toxicity in primary cells. The toxicity of MTA and DIG-MSK was low in mouse embryo fibroblasts and mononuclear blood cells obtained from healthy donors, and, in these cells, DIG-MSK showed significantly lower toxicity than MTA. These results are in agreement with *in vivo* studies were DIG-MSK has been well tolerated and no evidence of toxicity or cell death was observed in ovarian cells and other tissues [13].

Noteworthy, DIG-MSK retains a potent anti-tumor activity against cells obtained from different types of cancers, but ovarian cells were particularly sensitive to this drug. Similar to the parental MTA, DIG-MSK potently inhibited Sp1-mediated transcription in these ovarian tumor cells, and, given that the *Sp1* gene is autoregulated in a positive feedback, DIG-MSK was also a potent inhibitor of endogenous Sp1 expression. In correlation, DIG-MSK inhibited Sp1-regulated genes involved in the expression of key oncogenic processes, such as DNA repair (*BRCA2*), cell proliferation (*MYC*, *SRC*), telomere maintenance (*hTERT*) and cell survival (*BCL-XL*). Notably, p21^WAF1^/CDKN1A, which is involved in halting cells at G1 and G2/M phases, was induced in OVCAR-3 and IGROV-1, which correlated with a halt of the cell cycle and G1 arrest.

Of note, one of the genes strongly inhibited by DIG-MSK was *VEGFA*, which is a well-known Sp1-regulated gene in cancer [33,34]. In fact, it has been reported that MTA is a strong inhibitor of VEGF and it shows potent anti-angiogenic effects [14–18]. Nevertheless, we observed that DIG-MSK was more efficient than MTA in inhibiting key angiogenic genes. Thus, DIG-MSK was a strong inhibitor of VEGF pathway; but, contrasting with other anti-angiogenic drugs, DIG-MSK showed a dual effect inhibiting both *VEGFA* expression in cancer cells and *VEGFR1* and *VEGFR2* expression in ECs. Further, it also reduces the expression of other key angiogenic receptors, such as *FGFR*, *PDGFRA* and *PDGFRB*. Additionally, it up-regulates the expression of the anti-angiogenic genes encoding for angiostatin and tunstatin. The up-regulation of anti-angiogenic factors by MTA treatment has been previously described and it has been shown to exert anti-myeloma effect *in vivo* [17]. Conversely, MTA treatment increased *PDGFRA* mRNA expression and both drugs inhibited the expression of endostatin in HUVEC cells. However, these effects are cell-specific and could be linked to the specific characteristics of the cell line or with the transformation mechanisms of these cells. Further studies are needed to elucidate the role of DIG-MSK in the regulation of these genes in ECs. Nevertheless, the final effect of DIG-MSK treatment was a pro-apoptotic effect on proliferating microvascular ECs and the inhibition of the formation of capillary structures at non-toxic doses, which may be due to the effect of these angiogenic factors on the formation and stabilization of new blood vessels. Of note, MTA has a more limited effect on the inhibition of angiogenic factors and it was unable to inhibit the formation of capillary-like structures at non-toxic concentrations, suggesting that DIG-MSK has improved anti-angiogenic properties compared with the parental MTA.

Several anti-angiogenic drugs, mainly targeting VEGF and PDGF, have been successfully introduced in the anti-cancer therapy [5,6]. The majority of these drugs are highly specific and they are associated with low toxicity. However, many tumors become sooner or later refractory or resistant to VEGF blockade. Compensation by other angiogenic factors, such as FGF and angiopoietins, is a major factor that contributes to tumor resistance to VEGF blockade [35–37]. Contrasting the majority of drugs against angiogenesis, DIG-MSK shows pleiotropic effects on DNA targets that affect a wide range of Sp1-regulated genes. This array of anti-angiogenic and anti-oncogenic activities has important advantages and disadvantages. On one hand, they may limit the development of drug resistance. On the other hand, this multi-target activity may be associated with elevated toxicity, which may limit its therapeutic use. Notably, among many derivatives of MTA, DIG-MSK has been selected because it has showed a lower toxic profile than MTA, which highlights the potential of this drug as an efficient and safer anti-cancer agent.

## Supporting Information

S1 Fig Original and processed images of HMEC-1 tube formation using different concentrations of MTA and DIG-MSK. (PPTX)
