# Supplementary material for: Pleiotropic Anti-Angiogenic and Anti-Oncogenic Activities of the Novel Mithralog Demycarosyl-3D-ß-D-Digitoxosyl-Mithramycin SK (EC-8042)
Source: PLoS One. 2015 Nov 4;10(11):e0140786. doi: 10.1371/journal.pone.0140786 (PMC4633274; doi:10.1371/journal.pone.0140786)
Supplement: S1 Fig — (PPTX) [file pone.0140786.s001.pptx]

## Slide 1
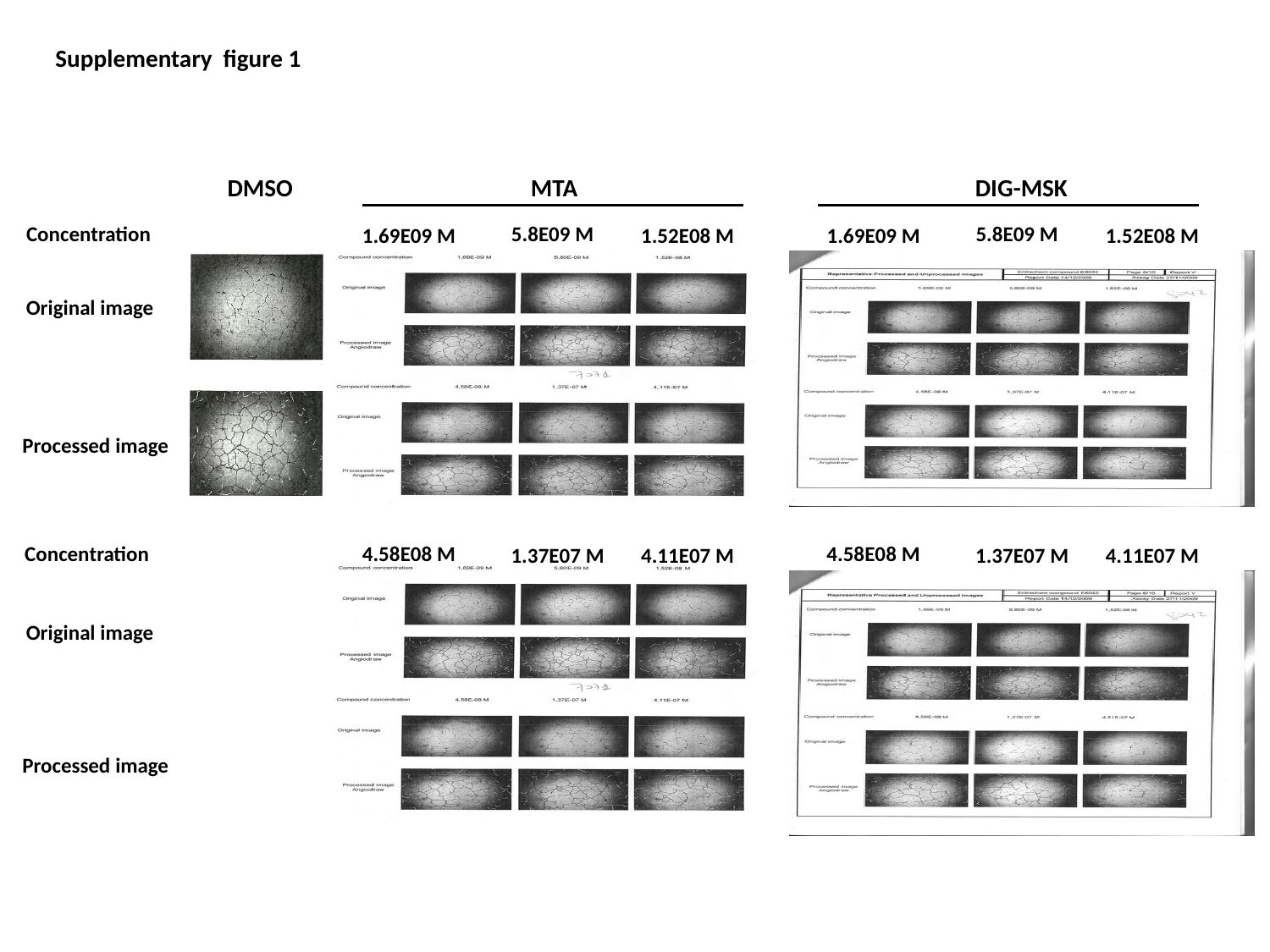

Supplementary figure 1
DMSO
MTA
DIG-MSK
Concentration
5.8E09 M
5.8E09 M
1.69E09 M
1.52E08 M
1.69E09 M
1.52E08 M
Original image
Processed image
Concentration
4.58E08 M
4.58E08 M
1.37E07 M
4.11E07 M
1.37E07 M
4.11E07 M
Original image
Processed image
